# Supplementary material for: Cinnamomum verum Bark Extract Mediated Green Synthesis of ZnO Nanoparticles and Their Antibacterial Potentiality
Source: Biomolecules. 2020 Feb 19;10(2):336. doi: 10.3390/biom10020336 (PMC7072335; doi:10.3390/biom10020336)
Supplement: Supplementary file 1 [file biomolecules-10-00336-s001.pdf]

Qualitative Report

File: C:\TurboMass\2019.PRO\Data\1-(19ES-1076).raw
Acquired: 02-Nov-19 03:30:54 AM — Printed: 06-Nov-19 10:39 AM
Description:
GC/MS Method: GC: METHOD-1.mth MS: METHOD-1.EXP — Page 1 of 1
Sample ID: 1-(19ES-1076) — Vial Number: 18

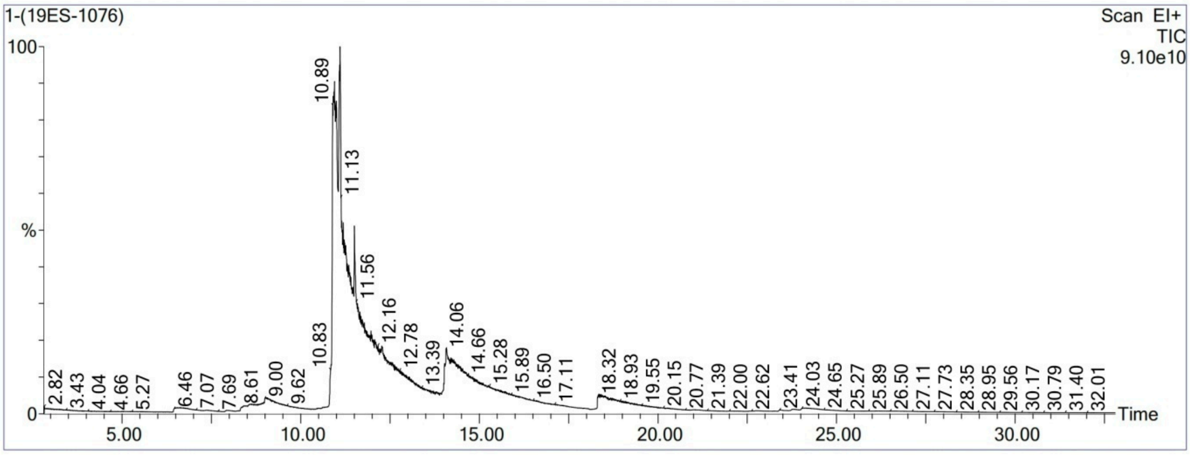

#	RT	Scan	Height	Area	Area %	Norm %
1	6.540	748	1,081,022,336	478,499,616.0	0.799	2.77
2	9.016	1243	2,043,318,400	841,907,840.0	1.406	4.87
3	10.942	1628	79,621,521,408	11,840,221,184.0	19.778	68.50
4	11.097	1659	88,098,717,696	17,285,081,088.0	28.873	100.00
5	11.497	1739	43,221,598,208	14,119,109,632.0	23.585	81.68
6	12.267	1893	12,679,901,184	7,115,513,856.0	11.886	41.17
7	14.073	2254	11,469,706,240	1,490,959,488.0	2.491	8.63
8	14.203	2280	9,007,903,744	5,490,076,672.0	9.171	31.76
9	18.360	3111	3,332,050,432	215,566,736.0	0.360	1.25
10	18.410	3121	3,226,878,208	608,523,072.0	1.016	3.52
11	18.670	3173	1,850,669,568	379,985,376.0	0.635	2.20

Figure S1. Chromatogram of *C. verum* bark hexane extract analyzed on GC-MS.

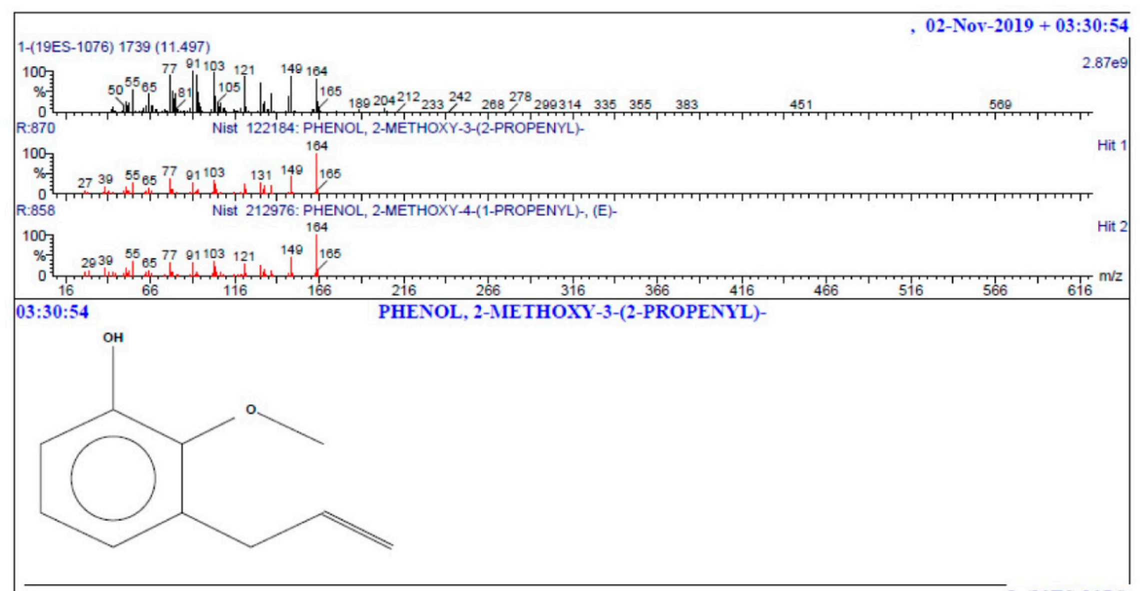

Hit	REV	for	Compound Name	M.W.	Formula	CAS
1	870	776	PHENOL, 2-METHOXY-3-(2-PROPENYL)-	164	C10H12O2	1941-12-4
2	858	772	PHENOL, 2-METHOXY-4-(1-PROPENYL)-, (E)-	164	C10H12O2	5932-68-3
3	856	764	EUGENOL	164	C10H12O2	97-53-0
4	851	747	PHENOL, 2-METHOXY-4-(1-PROPENYL)-	164	C10H12O2	97-54-1
5	840	750	3-ALLYL-6-METHOXYPHENOL	164	C10H12O2	501-19-9
6	839	756	PHENOL, 2-METHOXY-6-(2-PROPENYL)-	164	C10H12O2	579-60-2
7	837	737	PHENOL, 2-METHOXY-4-(1-PROPENYL)-, (Z)-	164	C10H12O2	5912-86-7
8	833	737	PHENOL, 2-METHOXY-6-(2-PROPENYL)-	164	C10H12O2	579-60-2
9	827	728	PHENOL, 2-METHOXY-4-(1-PROPENYL)-, (E)-	164	C10H12O2	5932-68-3
10	826	731	EUGENOL	164	C10H12O2	97-53-0
11	822	727	PHENOL, 2-METHOXY-4-(1-PROPENYL)-	164	C10H12O2	97-54-1
12	818	708	PHENOL, 2-METHOXY-5-(1-PROPENYL)-, (E)-	164	C10H12O2	19784-98-6
13	813	725	EUGENOL	164	C10H12O2	97-53-0
14	812	702	PHENOL, 2-METHOXY-4-(1-PROPENYL)-	164	C10H12O2	97-54-1
15	809	698	3-ALLYL-6-METHOXYPHENOL	164	C10H12O2	501-19-9
16	798	702	PHENOL, 2-METHOXY-4-(1-PROPENYL)-, (E)-	164	C10H12O2	5932-68-3
17	792	706	EUGENOL	164	C10H12O2	97-53-0

Figure S2. Retention time (RT) Library of *C. verum* bark hexane extract showing the eugenol content.

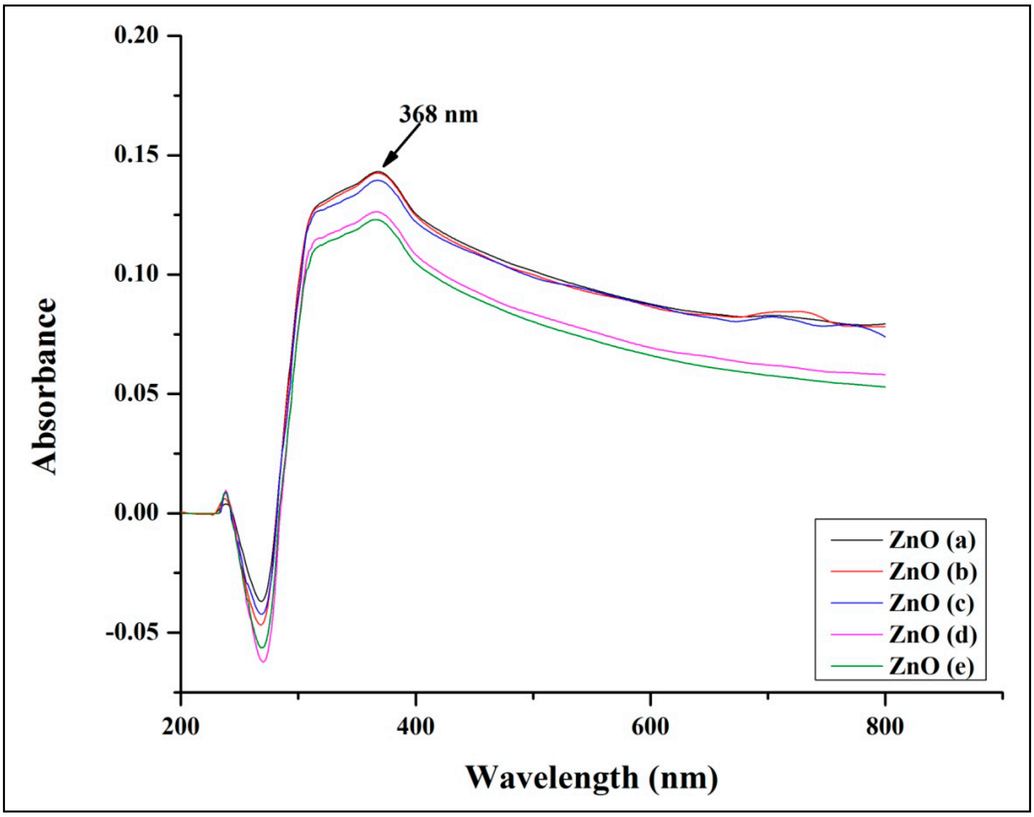

Figure S3. UV- visible spectra of various ZnO-NPs green synthesized using different concentration of *C. verum* hexane extract.

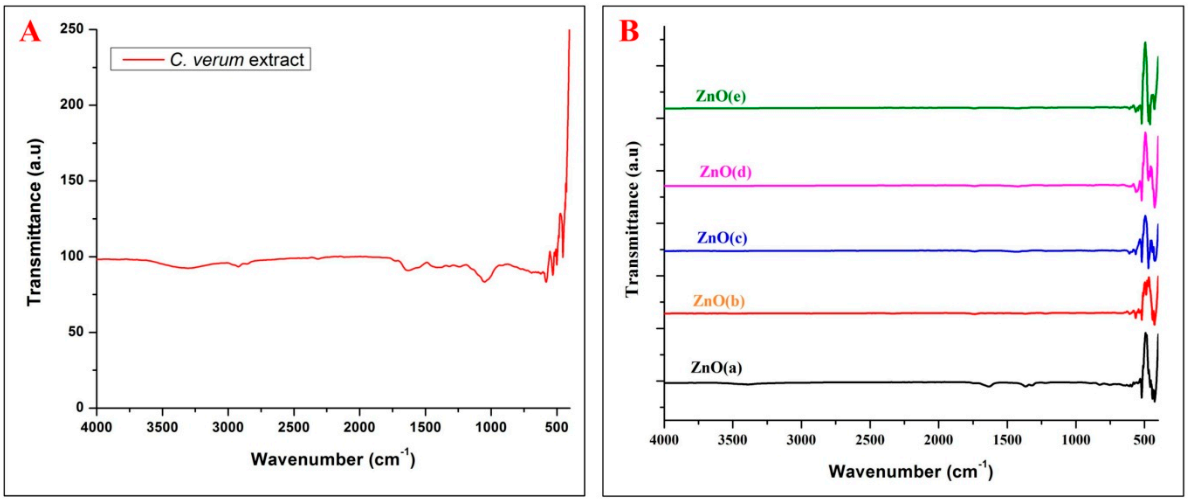

Figure S4. FT-IR Spectra of C. verum bark hexane extract (A) and its green synthesizesd ZnO-NPs (B)

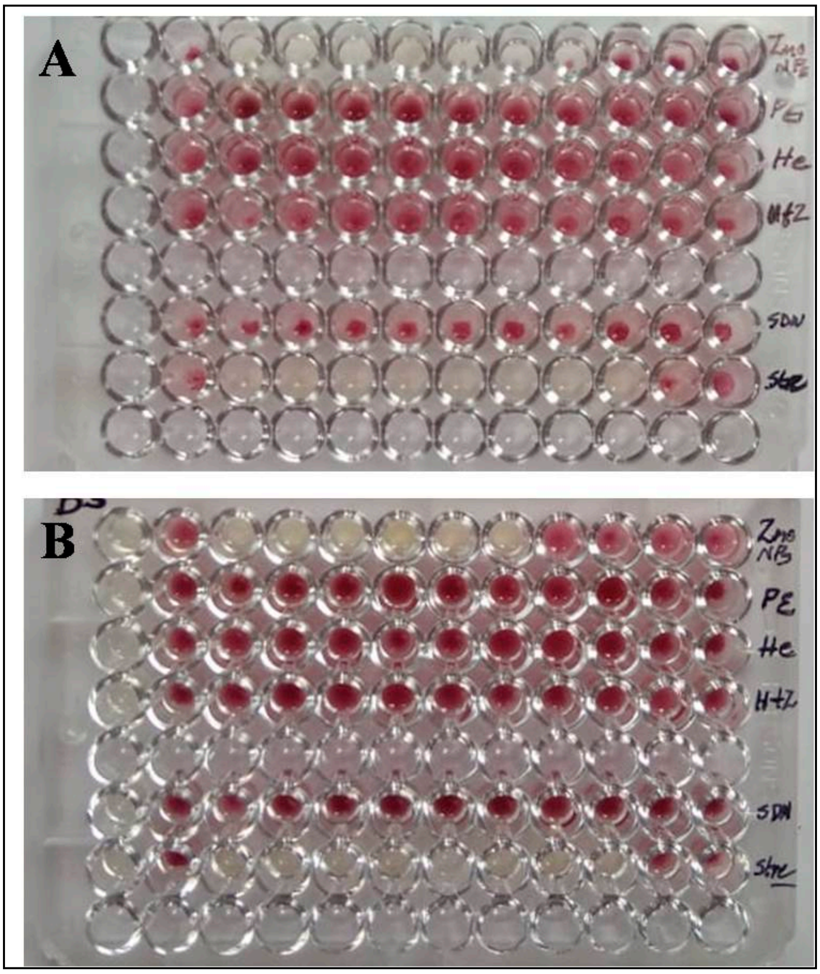

Figure S5. Minimum Inhibitory Concentration (MIC) of green synthesized ZnO-NPs from C. verum bark. hexane extract. A- *E. coli*; B- *S. aureus*. Row 1: ZnO-NPs; Row 2: Plant extract; Row 3: Hexane; Row 4: Hexane + Zinc nitrate hexahydrate; Row 5: Gap; Row 6: Sterile Distilled Water; Row 7: Streptomycin

**Well No.**											
**1**	**2**	**3**	**4**	**5**	**6**	**7**	**8**	**9**	**10**	**11**	**12**
Broth (100 µl)	Broth	Broth + Treatments (100 µl)	Broth + Treatments (100 µl)	Broth + Treatments (100 µl)	Broth + Treatments (100 µl)	Broth + Treatments (100 µl)	Broth + Treatments (100 µl)	Broth + Treatments (100 µl)	Broth + Treatments (100 µl)	Broth + Treatments (100 µl)	Broth + Treatments (100 µl)
-	-	2000 µg	1000 µg	500 µg	250 µg	125 µg	62.5 µg	31.25 µg	15.625 µg	7.8125 µg	3.9062 µg
	Culture (10 µL)	Culture (10 µL)	Culture (10 µL)	Culture (10 µL)	Culture (10 µL)	Culture (10 µL)	Culture (10 µL)	Culture (10 µL)	Culture (10 µL)	Culture (10 µL)	Culture (10 µL)
Incubated for 24 h at 37 ± 2 ºC											
After Incubation Absorbance was read at 620 nm											
After taking absorbance each well is added with 10 µL TTC to visualize the growth of bacterium (Red color indicate the viable bacteria)											
